# First Survey about Current Practices of Environmental Monitoring Programs within French Agri-Food Industries

**DOI:** 10.3390/biology11010089

**Published:** 2022-01-07

**Authors:** Juliana De Oliveira Mota, Pauline Kooh, Emmanuel Jaffrès, Hervé Prévost, Thomas Maignien, Nathalie Arnich, Moez Sanaa, Géraldine Boué, Michel Federighi

**Affiliations:** 1 French Agency for Food, Environmental and Occupational Health & Safety (ANSES), Risk Assessment Department, Maisons-Alfort, 94701 Paris, France; juliana.deoliveiramota@anses.fr (J.D.O.M.); pauline.kooh@anses.fr (P.K.); thomas.maignien@anses.fr (T.M.); nathalie.arnich@anses.fr (N.A.); moez.sanaa@anses.fr (M.S.); 2 SECALIM, INRAE, Oniris, 44307 Nantes, France; emmanuel.jaffres@oniris-nantes.fr (E.J.); herve.prevost@oniris-nantes.fr (H.P.); geraldine.boue@oniris-nantes.fr (G.B.)

**Keywords:** environmental monitoring programs, microbial risk, surfaces, survey, food safety, French food

## Abstract

**Simple Summary:**

Environmental monitoring programs (EMP) have become essential levers of action to ensure food safety. EMPs are already implemented in some food plants; however, knowledge about monitoring practices remains poorly disseminated. The present survey collected information on the monitoring practices of manufacturing environments in several sectors of the French agri-food industry. We observed that EMP strategies were based on a risk management approach. Practices are codified and transcribed in the HACCP system of some food manufacturers, which include hazards to be monitored, zones to be identified, surfaces to be sampled, tools for sampling and analysis, the number of samples collected, the frequency of sampling, monitoring contamination trends in microbial ecology, and corrective action plans for non-compliant results. EMP strategies are strongly related to food plant characteristics, and there is a lack of guidance. Therefore, a balance must be found between the harmonization of monitoring practices and the specificities of each site.

**Abstract:**

Food safety is a constant challenge for stakeholders in the food industry. To manage the likelihood of microbiological contamination, food safety management systems must be robust, including food and environmental testing. Environmental monitoring programs (EMP) have emerged this last decade aiming to validate cleaning–sanitation procedures and other environmental pathogen control programs. The need to monitor production environments has become evident because of recent foodborne outbreaks. However, the boundaries of environmental monitoring are not only limited to the management of pathogens but also extend to spoilage and hygiene indicators, microorganisms, allergens, and other hygiene monitoring. Surfaces in production environments can be a source of contamination, either through ineffective cleaning and disinfection procedures or through contamination during production by flows or operators. This study analyses the current practices of 37 French agri-food industries (small, medium, or large), reporting their objectives for EMPs, microbial targets, types, numbers and frequency of sampling, analysis of results, and types of corrective actions.

## 1. Introduction

Environmental monitoring programs (EMP) have emerged this last decade intended to validate cleaning–sanitation procedures’ efficiency and other environmental pathogen control programs through a range of sampling analyses to prevent contamination of the finished product by the environment [1]. Recent foodborne outbreaks such as the outbreak of listeriosis associated with processed meat in South Africa in 2017 [2] or with deli meats in Canada in 2008 [3] highlight the role of the production environment in the contamination of food products. Also, a recent outbreak associated with *Salmonella* Agona on infant formula was suspected to be from contamination of the processing environment of a French food plant [4]. This outbreak affected 39 infants: 37 in France, one in Spain, and one in Greece [5]. Consequently, the need to control the production environment has become evident. However, the boundaries of environmental monitoring are not only limited to the management of pathogens but also extended to spoilage organisms, indicator organisms, allergens, and hygiene monitoring. Surfaces in production environments can be a source of contamination, either through ineffective cleaning and disinfection procedures or through contamination during production by flows or operators.

The importance of the environment in food safety is such that competent authorities are beginning to provide a framework for its implementation, notably in Canada in 2004, the European Union in 2005, New Zealand in 2006 and 2020, and the United States of America in 2011 [6]. More particularly, in Europe, regulation (EC) No. 2073/2005 makes monitoring the production environment mandatory for *L. monocytogenes* in ready-to-eat foods and *Cronobacter* spp. in powdered infant formulae and powdered foods for special medical purposes intended for infants under six months of age. For these samples, the ISO/DIS 18,593 standard is used as a reference method. However, the frequency of sampling and the limits of microbiological criteria are only indicated for foodstuffs and not for environmental monitoring [7]. In addition, in France, law No. 2018/938 of 30 October 2018 (EGalim), stipulates compulsory notification by food business operators to the administrative authority if food could adversely affect human health as a result of contamination related to the production environment, such as facilities and equipment used to handle or store food or feed [8].

Food sectors groups [9,10,11] and governmental institutions [12,13,14,15] contribute to the development of guidelines to develop EMPs considering specific hazards of food products. However, knowledge of environmental monitoring practices by food business operators in France remains very limited because they are very dependent on the characteristics of each site (e.g., product type and production environment), not practiced by all, and relatively recent. The first objective of this survey was to discover if EMPs are performed by the French agri-food industry, by whom, for what purpose, and for how long before or after the change in regulations. Therefore, this study aims to identify, compare, and analyze the current practices of EMPs by food business operators to be compliant with EGalim law, focusing on microbial hazards and solid surfaces. Secondly, the aim of this survey is to share this information widely, while respecting the anonymity of the industrialists who answered the questionnaire, in order to strengthen EMP practices and help agri-food industries implement EMPs in plants where they are nonexistent.

## 2. Materials and Methods

### 2.1. Scope of the Online Survey and Definitions

An online survey was developed to identify current EMP practices in French agri-food industries (Figure S1). For a better understanding, first, key terms were defined. The French agri-food industry describes structures where food products are processed, packaged, or stored in France. The agri-food sector is a sector of activity corresponding to all the enterprises that produce or transform a type of product into industrial food. The same agri-food industry can produce food products from different sectors. The term “environment” used in this article corresponds to the food processing environment, which is defined in the standard NF EN ISO 18593:2018 as any element of a food plant that surrounds or could be in contact with the food product and is likely to represent a source of microbial contamination or recontamination such as the equipment, premises, or operators. Monitoring is defined by the standard NF V01-002:2005 as the implementation of a programmed series of observations or measurements to assess whether the food safety control measures are as efficient as intended. This article focuses on the microbiological monitoring of solid surfaces; therefore, fluids (e.g., air and water) and food products have been excluded. Sampling programs are plans designed to assess the levels of microbial contamination of the surfaces of the environment in order to implement corrective measures to prevent the contamination of food by microorganisms. Zones are delimited areas in the production environment, which can be defined according to specific criteria, such as the level of risk contamination or the processing steps. The sampling area defines the specific sampling localization (e.g., swab, wipes, etc.).

At first glance, the online survey allows us to know if EMPs were already implemented in the agri-food industries. Then, if EMPs were already applied, the survey enables us to identify and analyze strategies to establish, including a dedicated team, the type of microbial hazards researched, the zoning of the plant, the sampling program, the action plans based on results, and continuous improvement of the surveillance program. The questionnaire focused on monitoring microbiological hazards on solid surfaces. Therefore, food product and fluid monitoring, such as air and liquids, were not considered. Survey participants were aware of these requirements prior to completing the questionnaire.

### 2.2. Structure of the Online Survey

The questionnaire was developed with Google Forms and distributed by e-mail to food manufacturers without defined sectors through direct food business contacts or by agri-food association or competitiveness and innovation centers of the North-West of France: Valorial, Technocampus Aliment, Ligeriia, and the Association of Former Students of the Nantes-Atlantic National College of Veterinary Medicine, Food Science, and Engineering (Oniris). A webinar conducted by the authors of this study about the microbiological monitoring of production environments also promoted the questionnaire and allowed us to recruit additional agri-food industry participants.

The collection of responses to the questionnaire took place from 15 September 2020 to 27 November 2020. The questionnaire was organized into 40 questions under six sections:

- Information on the subject of the questionnaire and its relevance;
- Description of the respondent;
- The pre-analytical phase of the surface control plan corresponding to the zoning definition, the choices about the types of materials and surfaces to be sampled, the methods and tools for sampling, the periodicity of these, and the microbiological targets to be sought;
- The analytical phase consisting of the analysis of the surface samples;
- The post-analytical phase concerning the interpretation of the results and the implementation of an action plan;
- The project’s follow-up, which asked whether the participant wanted to be included in the future of the project.

In order to improve the duration of the questionnaire and optimize the number of responses, the survey was designed with three different question formats: multiple choice, checkboxes, or short answers. The number of mandatory answers by the food industry participant was limited to the name of the company and the location of the food plant. The authors have guaranteed the confidentiality of all individual responses, and the full version of the survey is available in the Supplementary Material.

### 2.3. In-Depth Interview with Food Manufacturers

In the last section of the survey, the participants were invited to deepen their answers to the questionnaire through a telephone, video, or face-to-face interview. The questions asked during this interview were specific to each participant, based on their answers to the questionnaire, in order to get closer to the reality of practices for specific cases. Interviews from six French agri-food industries were recorded with the permission of the interviewees in order to transcribe the answers as faithfully as possible. For the online questionnaire, the authors have guaranteed the confidentiality of all individual responses given in the personalized interviews.

## 3. Results

### 3.1. General Description of the Participants of the Online Survey

Thirty-seven French agro-food industries participated in the questionnaire. All of the participants were included in the analysis of the survey: 68% (n = 25) of the respondents belonged to small or medium companies (between 10 and 249 employees), 22% (n = 8) to mid-size companies (between 250 and 4999 employees), 5% (n = 2) to very small (less than 10 employees, and 5% (n = 2) to big companies (equal or over 5000 employees). The majority of the agri-food industries have been active for more than 30 years (43%, n = 16).

**Figure 1:**
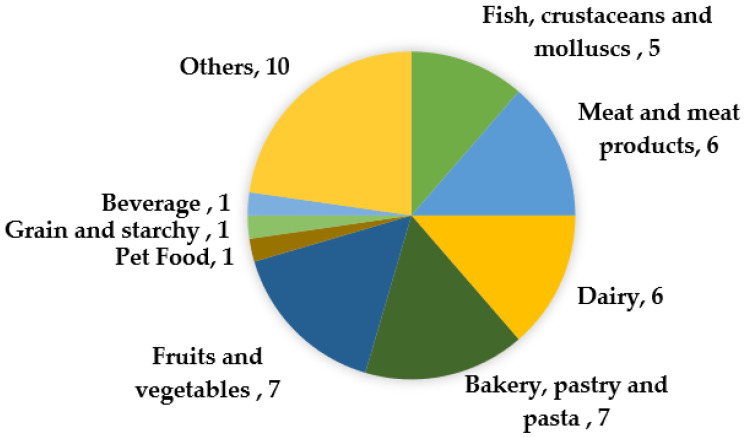
Number of surveyed agri-food industry respondents considered in the study by sector of activity.

The respondents’ sector of activity showed a large diversity (Figure 1). The first two sectors of activity represented in the survey were “fruit and vegetable products” (n = 7) and “bakery and pasta products” (n = 7). These were followed by “dairy products” and “meat and meat products” with six participants each, followed by “fish, crustacean, and mollusc products” (n = 5), “drink products” (n = 1), “grain and starch products” (n = 1), and “animal feed products” (n = 1). Ten respondents declared producing other types of food (not specified). Five respondents reported having two or more sectors of activity in the same agri-food industry.

Almost all agri-food industry respondents, except for one, were already interested in EMP practices. In addition, 29 of the respondents have been interested in EMPs for more than two years.

Twenty-nine respondents declared that they have a team in charge of the EMP integrated into the quality department, three of which stated that they also involved operators from the production department.

The majority of the respondents stated that they implemented an EMP to validate cleaning and disinfection procedures (89%, n = 33), detect the presence of pathogens in the production environment (62%, n = 23), and monitor the microbial population of the plant in order to detect abnormal variations (51%, n = 19). Since EMP procedures can cover several goals simultaneously, most of the time, the objectives of this approach are multiple and overlapping, which is why for 38% (n = 14) of the respondents, their EMP goals corresponded to the three goals described above (validate cleaning and disinfection procedures, detect the presence of pathogens, and monitor the microbial population of the plant). Four agri-food industry respondents declared that they implemented EMPs to validate cleaning and disinfection procedures and monitor the microbial population of the plant. Six agri-food industry respondents used EMPs to validate cleaning and disinfection procedures and detect the presence of pathogens in the production environment. A dairy manufacturer declared that their EMP aimed to monitor the microbial population in the plant and to detect pathogenic microorganisms, but not to validate the cleaning and disinfection procedures. One-third of the respondents reported having only one goal: to validate the cleaning and disinfection procedures (24%, n = 9) or to detect pathogenic microorganisms (5%, n = 2).

From the interviews, it was observed that EMPs were often formalized within the sanitary control program of the food factories, covered by pre-requisites programs (Good Hygienic Practices) or, sometimes, in HACCP plans even if not designed to plan EMPs at first glance.

### 3.2. Microorganisms Monitored in Environmental Processing Surfaces

The survey revealed that *Listeria* spp., including *Listeria monocytogenes*, were the most monitored microorganisms by the French agri-food industry respondents (54%, n = 20), followed by *Salmonella* spp. (46%, n = 17), *E. coli* (16%, n = 6), *Staphylococcus aureus* (11%, n = 4), and *Cronobacter* spp. (5%, n = 2). The main groups of microorganisms monitored were Total Aerobic Count 30 °C (78%, n = 29), total coliforms (49%, n = 18), Enterobacteria (46%, n = 17), mould (43%, n =1 6), and yeast (41%, n = 15).

*Listeria* spp. were mainly monitored in the processing and preservation of meat and the preparation of meat products industries, and the processing and preservation of fish, crustaceans, and molluscs industries. *Salmonella* spp. were mainly monitored in the dairy industry environments as *Cronobacter* spp., which were only monitored in the dairy products industries. Total Aerobic Count 30 °C was monitored by all French agri-food industry respondents from the following processing and preservation sectors: fruits and vegetables; meat and meat products; fish, crustaceans, and molluscs; drinks; grain and starchy products; and beverages (Table 1).

Four French agri-food industries reported testing for the presence of biofilms as part of their monitoring plan.

### 3.3. Zoning Concept in Environmental Monitoring Programs

The majority of the French agri-food industries (n = 31) declared that they identified different zones in their EMPs. The definition of zones was highly variable from one plant to the next. Ten respondents declared that the zones corresponded to specific activities in a particular area of the plant (e.g., administration area, production area, packaging area, and storage area). Nineteen French agri-food industry respondents declared that they defined zones according to the vulnerability of the product to contamination, which corresponds to a specific area in the plant where a production step is executed or with the proximity of a bare product to a surface (e.g., material in contact with the product, critical environment, and non-critical environment). The plan for monitoring surfaces in the production environment is mainly related to zoning (n = 29, 78% of all the respondents) for those who responded to the related question (n = 34).

### 3.4. Sampling Tools

The main tools for the sampling of production areas were wipes (n = 19), agar slides (n = 16), contact agar plates (n = 9), swabs (n = 9), and a bio-collector (n = 1).

To detect the presence of biofilms, the French agri-food industries concerned (n = 4) use products available on the market, such as biofilm stain detectors.

### 3.5. Sampling Moments and Frequency

**Figure 2:**
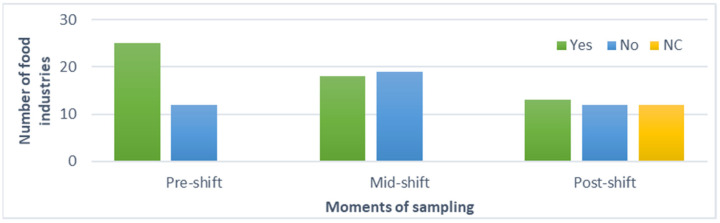
Sample collection moments identified by surveyed food facilities. The same agri-food industry can sample at different moments of the production cycle. NC: Not communicated.

Sampling can be processed before (pre-shift), during (mid-shift), or after (post-shift) the production cycle. In addition, this procedure can be done several times during the production shift. Half of the French agri-food industries sampled pre-shift (68%, n = 25), 49% during the production cycle (n = 18), and 35% post-shift (n =13) (Figure 2). Seventeen food industries reported sampling at least two different periods.

Pre-shift sampling was mainly carried out monthly (27%, n = 10) or weekly (24%, n = 9) and less often several times a day or a month (3%, n = 1). Sampling during the production cycle was mainly performed weekly (22%, n = 8) and monthly (14%, n = 5). Less frequently, sampling procedures were performed several times a month (3%, n = 1) or bimonthly (3%, n = 1). Finally, for post-shift sampling, 12 French agri-food industries did not answer this question. Sampling was declared to mainly proceed monthly (16%, n = 6) or once a day (8%, n = 3), and less frequently weekly (3%, n = 1), several times a week (3%, n = 1), or biannually (3%, n = 1).

### 3.6. Number of Environmental Samples

Thirty French agri-food industries gave information on the number of areas sampled. A mean of 188 areas are sampled; however, this average is not very representative because the number of identified surfaces varies from one factory to the next (standard deviation: 290).

**Figure 3:**
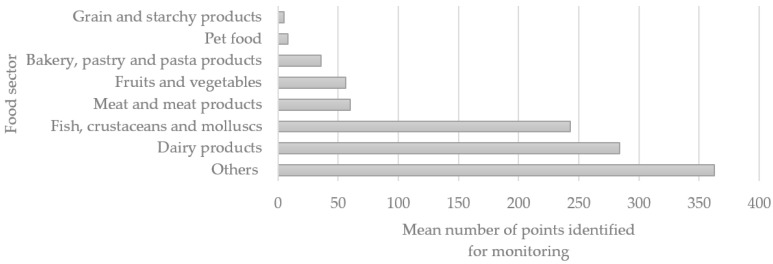
Mean number of sampling points in environmental monitoring in surveyed food facilities.

After the other types of products, dairy product manufacturers collected the highest number of samples from environmental surfaces (mean = 284), followed by the seafood sector and meat and meat product industries. Grain and starchy products manufacturers declared the lowest number of environmental samples (Figure 3).

It was observed that more samples are taken, on average, from the environment for companies with plants with larger surface areas and higher tonnage of food products. However, as the number of responses was low, a conclusion about the statistical relationship was not achievable.

### 3.7. Analysis of Environmental Samples

The analysis of environmental samples is carried out by the plant’s internal laboratory (49%, n = 18), an external laboratory (41%, n = 15), by the operator in charge of sampling (14%, n = 5), or by the quality manager (3%, n = 1). For 13 French agri-food industries, analyses are carried out by at least two different means, from which seven include analysis from an internal and external laboratory, and six include analysis from an internal and/or external laboratory and by the operator in charge of sampling or the quality manager. Four respondents stated that the operator in charge of sampling only carried out analyses. Thirty-five agri-food industries responded to the question about the methods of analysis of the samples, all of which stated that they practice cultural techniques. Three reported proceeding with molecular analyses, and one reported conducting immunological analyses.

### 3.8. Management of the Environmental Sample Analysis Results

When the teams in charge of the EMP were in possession of the results of the sample analyses, they could interpret them using pre-established decision grids (49%, n = 18) or not (46%, n = 17). Interviews revealed that the thresholds for non-pathogenic microorganisms were determined either by monitoring contamination trends in the plant or by a benchmark with other plants in the same food sector. In addition, the majority of French agri-food industries declared that they follow the trends of microorganism contamination in the environment of their production plants (89%, n = 33) and that tracking trend results affected the monitoring plan (84%, n = 31).

The interpretation can be the presence or absence of the microorganism (24%, n = 9) mainly for *Listeria*, *Salmonella,* and *Cronobacter* species or based on the enumeration of colony-forming units and comparing them to pre-established thresholds. Only a few companies (n = 11) agreed to communicate their grid of interpretation of the sampling results, mainly in the bakery, pastry, and pasta sectors and dairy sectors, by either answering the questionnaire or providing an interview (Table 2).

The interviews with the agri-food industries (16%, n = 6) revealed that for each threshold or class, an action plan was associated. When the result is in compliance, production and environmental control procedures remain unchanged, and the frequency and number of controls could be reduced in the future, if this reduction guarantees control of food safety, after decisions made during annual or biannual meetings by plant management or the HACCP team (14%, n = 5). In cases of non-compliance, an investigation of the source of contamination was done, and cleaning and disinfection procedures were reinforced. If the surface is considered at high risk of product contamination and the microorganism is pathogenic (ex. *Salmonella* spp.), the food product lot might be blocked until the product test result meets the requirements. The non-compliant surfaces were then controlled during the next production cycles or the next scheduled control session.

## 4. Discussion

To our knowledge, this study is the first in France that investigated and analyzed the monitoring practices of production environments within the agri-food industries, following the implementation of the French law EGalim No. 2018/938. This law requires a declaration to the authorities if agri-food industries find that a food product is likely to be harmful to human health from contamination associated with the food production environment [8].

Despite the importance of monitoring microbial contamination of the surfaces in the production environment, the practices are poorly disseminated because they are highly dependent on the characteristics of each production site, such as the type of food produced, the size of the plant, and the number of operators [16,17]. Therefore, there is a lack of benchmarks, particularly for microbiological criteria suitable to food manufacturing surfaces. The purpose of this study was to begin providing insights into EMP practices, in particular for microbiological monitoring of surfaces in production environments. An online survey followed by individual interviews of the respondents who accepted was carried out and described in this article.

Several food sectors with different sizes in terms of surface area and tons of product per year were represented in this study. However, the number of French agri-food industry respondents was insufficient to provide robust statistical results that would significantly represent practices by activity sector and size. Indeed, the dissemination of the survey by specific networks located in North-Western France (Valorial, Technocampus Aliment, Ligeriia, and the Association of Former Students of Oniris) mobilized agri-food industries from this localization and fewer French agri-food industries from other regions.

Nevertheless, the examples described in this study are highly valuable since they will subsequently help the implementation of EMPs for plants that do not yet have or that are in the process of building their EMP or by improving existing practices.

Through the online survey and individual interviews conducted afterwards, it appears that the importance of these practices is undeniable. Indeed, the survey highlighted that the majority of the French agri-food industries surveyed (36 over 37) were interested in EMP practices for more than two years (29 over 37). In addition, they declared that they implemented this monitoring program mainly to validate cleaning and disinfection procedures (n = 33, 89%), detect the presence of pathogens in the production environment (23%, n = 62%), and monitor the microbial population in the plant to detect suspicious variations in the microbiological population (n = 19, 51%), which corresponds to the main goals described in the literature for EMP implementation [17,18].

From the survey, it was observed that EMPs were often formalized within the sanitary control program of food factories, covered under pre-requisite programs (Good Hygienic Practices) or, sometimes, in the HACCP plan. However, in Europe, it is mandatory to use the principles behind the HACCP method, not to use HACCP method. Interestingly, none of the 12 tasks of the HACCP method is dedicated to EMPs [19]. From this point of view, EMPs appear to complement the HACCP approach.

European regulation (EC) No 2073/2005 requires detecting the presence of pathogenic bacteria in the processing environment and the corresponding processed product. However, the only specified pathogens cited in this regulation are *Listeria monocytogenes* in ready-to-eat foods and *Cronobacter* spp. in powdered infant formulae or powdered foods for special medical purposes intended for infants under six months of age [7]. Our survey highlighted that French agri-food industries go further than the regulatory requirements in terms of monitoring microorganisms. Indeed, four main types of microorganisms were monitored by French agri-food industries as part of their EMPs: pathogens, index, indicators, and spoilage organisms. The most searched pathogenic microorganisms were *Listeria monocytogenes*, *Salmonella* spp., *Cronobacter* spp., and *Staphylococcus aureus*. Depending on the food sector, *E. coli* was researched both as a pathogen and/or an indicator microorganism.

The persistence of these pathogens in food processing environments has been demonstrated. Moreover, numerous foodborne outbreaks have been associated with contamination from food processing environments such as *Listeria monocytogenes* in ready-to-eat foods, *Salmonella* spp. and low moisture foods, and *Cronobacter* spp. and dairy powder [20,21,22,23].

Several guidelines recommend monitoring indicator organisms [10,15], which may include surrogates and index organisms in EMPs [24]. These may suggest the potential presence of pathogens [25] and allow the detection of a potential risk of contamination (e.g., increase of the microbial population over time). The most common indicator organisms, corresponding to a variety of microorganisms present in the environment [24], were Total Aerobic Count 30 °C, total coliforms, *E. coli*, and Enterobacteria. The major index organism corresponded to *Listeria* spp. as a marker to detect the possible presence of *Listeria monocytogenes* due to similar ecological characteristics [17,24], as recommended by several guidelines [10,15,17]. Finally, yeast and moulds were monitored and could be assimilated to spoilage organisms, notably in the bakery, pastry, and pasta sectors. Few French agri-food industries declared that they monitor the presence of biofilm; however, it is increasingly recognized that meat pathogens and spoilage organisms develop predominantly in this aggregated and adhered bacterium to a surface form rather than in their planktonic form [26]. The issue is that biofilms have higher resistance to disinfectants and, therefore, become a reservoir of organisms that can be constantly released into food [27,28].

To monitor these microorganisms, guidelines recommend determining different zones in the food facility [9,14,29] and establishing a sampling and testing strategy for each zone. The delimitation and definition of the zones considered in the EMP appear to be a central element of reflection by the French agri-food industries and must be based on risk [1], notably through the assessment of the risk by modelling and a consideration of the agri-food industries. As an example, the New South Wales Government guidelines defined two zones: food contact surfaces and non-food contact surfaces. The Food Drugs Administration recommended four zones: from zone 1 with the highest risk of contamination because the surfaces are in contact with the product (e.g., slicers), up to zone 4 with the lowest risk of contamination (e.g., office and employee break areas) [30]. The plan for monitoring surfaces in the production environment was declared to be mainly related to zoning (n = 29, 78%). However, it was shown in our study that the definition of zones was not the same for all French agri-food industries that responded to the survey. Indeed, zones can be identified according to the level of risk contamination, the step of the process, or the nature of the food.

The number of samples to be taken and the frequency of sampling are other very important criteria to consider in an EMP and are intimately related to the zones. The method for determining the number and frequency of samples needed to ensure food safety is risk-based but not clearly established because it is strongly related to the food plant characteristics, historical contamination, and resources [31]. As a result, it was observed that more samples were taken from the environment, on average, for companies with larger surface areas and higher tonnage of food products. On the other hand, the sampling frequency seems to be somewhat related to the vulnerability of the product to contamination. Indeed, surface monitoring was observed to be more frequent for animal products, with weekly sampling, compared to monthly sampling for vegetable products. Interviews revealed that sampling frequency was also related to the results from previous monitoring sessions, as recommended by existing EMP guidelines [10]. Little information is available on the number of samples to perform and the frequency per type of food product. For example, guidance documents recommend sampling at least three to five areas on food contact surfaces per line for *Listeria* spp. surveillance with variable frequency based on risk [15]. In *Salmonella* spp. surveillance, the Almond Board of California recommends performing a weekly sampling of the most at-risk zones to a monthly sampling of the least risky zones (e.g., an office desk), with an increasing number of samples as the risk of contamination increases [9].

After sampling, the analysis was reported to be performed by the operator in charge of sampling, by the internal laboratory of the plant, or by an external laboratory. The majority of the analysis methods were by cultural approaches, which are the most common methods, notably listed as reference methods in annex I of the European Regulation No 2073/2005. Molecular subtyping methods (e.g., PFGE and MLVA) were used for the identification of contamination sources within production facilities [32]. Recent technological advances in Whole Genome Sequencing can improve the analyses and facilitate the tracking of microorganisms within processing facilities, but, for now, these technologies remain limited to specialized laboratories [33].

After analysis, the team in charge of the EMP must manage the results and set up pre-defined corrective actions. The interviews revealed how difficult it is for French agri-food industries to determine limits for each microorganism or group of microorganisms due to the absence of regulations or examples on microbiological criteria for surfaces in production environments. Our study allowed compiling different classes and the associated thresholds for several microorganisms and several food sectors. Even if the number of examples is limited and more knowledge from other French agri-food industries would be beneficial, this study will enable us to start a benchmark of the thresholds and decision grids and adjust them afterward. Otherwise, the majority of French agri-food industries declared that they follow the trends of microorganism contamination in the environment of their production, and the interviews revealed that the thresholds were determined according to contamination trends, which is the recommended method by the guidelines. Indeed, following the trends of contamination over time increases knowledge about environmental conditions and the effectiveness of contamination risk prevention [14,17].

Our study identified practices for monitoring production environments, which are undeniably levers to improve food safety, in addition to food product analyses. Indeed, this environmental monitoring can be a tool for downstream monitoring, allowing the identification of an increased risk of food product contamination before it appears. Moreover, environmental monitoring can also lead to understanding the origin of contamination of a food product, when one appears, by analyzing the evolution of the microorganism population in the plant, thanks to the traceability of the sampling results for a period of time, and to proceed to corrective measures in terms of procedures for cleaning and disinfection or the structure and the architecture of plant installations.

Although an online survey is less comprehensive than a one-on-one interview, the survey provided information from a wider range of industry participants. The subsequent interview, for the French agri-food industries that accepted, provided more details on practices and shed light on some of the points raised in the online questionnaire.

## 5. Conclusions

This first survey on EMP practices in food industries in France demonstrated the interest in EMP practices by almost all participants and that this practice was in place for a large part of the agri-food industries well before the EGalim law. It has been demonstrated that EMP strategies and practices are often codified and transcribed in the HACCP system of French agri-food industries included in the survey, but the approach is very site-dependent and must take into account the product manufactured, the processes used, the volume of production, and the location. EMPs were applied not only to detect pathogens on surfaces but also to follow microbial indicators in a population over a period of time, with recurrent sampling frequency in specific zones of the production environment. However, there is a lack of guidance, as expressed by the surveyed French agri-food industries. A balance must be found between harmonization and codification of monitoring practices and the specificities of each site. The French agri-food industries must, therefore, be very active in defining a monitoring strategy.

In the future, surveys such as this should be expanded to provide more reliable and site-specific data in order to provide examples of agri-food industry practices and leads for implementing EMPs and codifying practices in order to help French agri-food industries apply effective EMPs.

## Figures and Tables

**Table 1:** Recurrence of monitoring microorganisms or groups of microorganisms within the framework of the Environmental Monitoring Programs by the French agri-food industries surveyed. The same agri-food industry corresponds to several food sectors. The results are expressed as the number of agri-food industries that responded to monitoring the hazard, and the values in brackets correspond to the percentage of agri-food industries within the same food sector concerned by the hazard being monitored.

Sector of Activity	*Listeria* spp.	*Listeria* *Monocytogenes*	*Salmonella* spp.	*Escherichia coli*	*Cronobacter* spp.	*Staphylococcus Aureus*	Total Aerobic Count 30 °C	Total ColiForms	Enterobacteria	Yeasts	Mould	None
Bakery, pastry, and pasta	2	2	2	3	-	2	6	4	3	5	5	-
	(29%)	(29%)	(29%)	(43%)		(29%)	(86%)	(57%)	(43%)	(71%)	(71%)	(-%)
Beverage	-	-	-	-	-	-	1	-	-	1	1	-
							(100%)			(100%)	(100%)	
Dairy	2	2	6	-	2	1	2	1	5	3	3	-
	(33%)	(33%)	(100%)		(33%)	(17%)	(33%)	(17%)	(83%)	(50%)	(50%)	
Fish, crustaceans, and molluscs	4	2	1	-	-	-	5	5	1	-	-	-
	(80%)	(40%)	(20%)				(100%)	(100%)	(20%)			
Fruits and vegetables	2	1	1	1	-	-	7	3	1	1	2	-
	29%	(14%)	(14%)	(14%)			(100%)	(43%)	(14%)	(14%)	(29%)	
Grain and starchy	-	-	-	-	-	-	1	1	-	1	1	-
							(100%)	(100%)		(100%)	(100%)	
Meat and meat products	5	4	3	1	-	-	6	5	2	1	-	-
	(83%)	(67%)	(50%)	(17%)			(100%)	(83%)	(33%)	(17%)		
Pet food	-	-	1	-	-	-	-	-	1	-	-	-
			(100%)						(100%)			
Other	4	6	4	1	-	1	9	5	4	4	4	1
	(40%)	(60%)	(40%)	(10%)		(10%)	(90%)	(50%)	(40%)	(40%)	(40%)	(10%)

**Table 2:** Grid of decision for compliance in microbiological monitoring on surfaces reported by the agri-food industries surveyed. NA: Not Available.

Microorganism	Limits (ufc)	Compliance	Food Sector
*Listeria* spp.	Absence	Compliant	Bakery, pastry, and pasta (n = 2)
	Presence	Non-compliant	
*Listeria monocytogenes*	Absence	Compliant	Bakery, pastry, and pasta (n = 2) Others (n = 2)
	Presence	Non-compliant	
*Listeria monocytogenes * (non-contact with food)	0–10 ufc	Compliant	Bakery, pastry, and pasta (n = 2)
	>10 ufc	Non-compliant	
*Salmonella* spp.	Absence	Compliant	Bakery, pastry, and pasta(n = 2) Dairy (n = 2)
	Presence	Non-compliant	
*Cronobacter* spp.	Absence	Compliant	Dairy (n = 1)
	Presence	Non-compliant	
Coliforms	0–10 ufc	Compliant	Bakery, pastry, and pasta (n = 2)
	>10 ufc	Non-compliant	
Coliforms	Absence	Rate = 3	Others (n = 1)
	Presence	Presence = 0	
Enterobacteria (equipment surface)	0–100 ufc	Compliant	Dairy (n = 2)
	>100 ufc	Non-compliant	
Enterobacteria	<10 ufc	NA	Dairy (n = 1)
	10–150 ufc	NA	
	>150 ufc	NA	
Enterobacteria (hands)	0–1 ufc	Compliant	Bakery, pastry, and pasta(n = 2)
	2–5 ufc	Tolerable	
	>5 ufc	Non-compliant	
Total Aerobic Count 30 °C	0–2 ufc	Very good	Bakery, pastry, and pasta (n = 1)
	3–10 ufc	Good	
	11–32 ufc	Tolerable	
	33–100 ufc	Poor	
	101–317 ufc	Bad	
	>317 ufc	Very bad	
Total Aerobic Count 30 °C	0–15 ufc	Good	Others (n = 1)
	15–45 ufc	Tolerable	
	>45 ufc	Bad	
Total Aerobic Count 30 °C	0–10 ufc	Rate = 3	Others (n = 1)
	11–20	Rate = 2	
	21–30	Rate = 1	
	>30	Rate = 0	

## Data Availability

No data available.

## Supplementary Materials

The following are available online at https://www.mdpi.com/article/10.3390/biology11010089/s1, Figure S1: Online Survey form.
