# Supplementary material for: First Survey about Current Practices of Environmental Monitoring Programs within French Agri-Food Industries
Source: Biology (Basel). 2022 Jan 7;11(1):89. doi: 10.3390/biology11010089 (PMC8772832; doi:10.3390/biology11010089)
Supplement: Supplementary file 1 [file biology-11-00089-s001.zip › biology-1512467-supplementary.pdf]

Supplementary material

First survey about current practices of environmental monitoring programs within French agri-food industries

Juliana De Oliveira Mota[1,2], Pauline Kooh[1], Emmanuel Jaffrès[2], Hervé Prévost[2], Thomas Maignien[1], Nathalie Arnich[1], Moez Sanaa[1], Géraldine Boué[2], Michel Federighi[2,*]

[1] French Agency for Food, Environmental and Occupational Health & Safety (ANSES), Risk Assessment Department, 14 rue Pierre et Marie Curie, 94701 Maisons-Alfort, France; (J.D.O.M) juliana.deoliveiramota@anses.fr, (T.M.) thomas.maignien@anses.fr, (N.A) nathalie.arnich@anses.fr, (P.K.) pauline.kooh@anses.fr, (M.S.) moez.sanaa@anses.fr

[2] INRAE, Oniris, SECALIM, 44307 Nantes, France; (G.B) geraldine.boue@oniris-nantes.fr, (E.J.) emmanuel.jaffres@oniris-nantes.fr, (H.P) herve.prevost@oniris-nantes.fr, (M.F) michel.federighi@oniris-nantes.fr

* Correspondence: michel.federighi@oniris-nantes.fr

Figure S1: Online Survey form

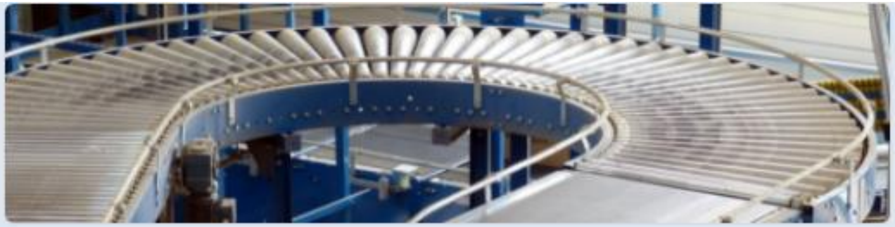

Environmental Monitoring: State of the Art and Practices in Agri-Food Industries

Recently, the EGAlim law requires the warning of the administrative authorities when the food actors observe that the "premises, installations and equipment used for the handling or the storage of foodstuffs and animal feed are likely to make products harmful to the environment. human health ".

The implementation and methodologies for monitoring surfaces in the production environment, as well as the interpretation and use of the results, are the responsibility of stakeholders in the agrifood sector.

The purpose of this questionnaire is to list and analyze data on industrial practices with regard to an approach to control microbiological hazards in the food production environment, in particular on open solid surfaces.

NB In this project, the term "environmental" concerns everything that surrounds the product during its production (surfaces, air, water). In this questionnaire, only the monitoring of solid surfaces is concerned.

Each site has its specificities. In order to identify the practices as accurately as possible, it would be preferable for each factory to answer this questionnaire. If not, please indicate below how many sites are represented by the following responses:

Your answer

Participant Description

1. Company name (will be anonymized) *

Your answer

2. City and department of location *

Your answer

3. Please select your business sector

- ☐ The processing and preservation of meat and the preparation of meat products
- ☐ The processing and preservation of fish, crustaceans and molluscs
- ☐ Processing and preserving of fruits and vegetables
- ☐ The manufacture of vegetable and animal oils and fats
- ☐ The manufacture of dairy products
- ☐ Working the grains, making starch products
- ☐ Manufacture of bakery and pasta products
- ☐ The manufacture of animal feed
- ☐ The manufacture of beverages
- ☐ Other manufactures

4. Type of food products produced

- ☐ Unit raw products
- ☐ Assembled raw products (mixed salads, mixture of raw vegetables, etc.)
- ☐ Unit cooked products
- ☐ Assembled cooked products (ready meals, mixed salads, sandwiches, etc.)
- ☐ Other :

5. Company size

- ◯ Large company (≥5000 employees)
- ◯ Mid-size company (between 250 and 4,999 employees)
- ◯ Small and medium-sized business (between 10 and 249 employees)
- ◯ Microenterprise (less than 10 employees)

6. Number of employees in the plant concerned (not compulsory)

Your answer

7. Surface area of the plant concerned

- ◯ Less than 1000 m²
- ◯ Between 1000 m² and 5000 m²
- ◯ More than 5000 m²

8. Tons produced per year by the plant concerned (not compulsory)

- ◯ <1000
- ◯ 1000 - 10,000
- ◯ 10,001-100,000
- ◯ > 100,000

9. Age of plant

- ◯ Less than 10 years
- ◯ 10-20 years
- ◯ 20-30 years
- ◯ Over 30 years

10. Has there been a factory renovation in the last 10 years?

- ◯ Yes
- ◯ No

11. Are you interested in controlling the production environment?

- ◯ Yes, for less than 2 years
- ◯ Yes, for more than 2 years
- ◯ No

12. Is there any zoning in your business?

- ◯ Yes
- ◯ No

13. Name the different zones

Your answer

14. What is the main criterion which led you to differentiate the different zones? (not required)

Your answer

15. Is the surface monitoring plan in the production environment linked to zoning?

- ◯ Yes
- ◯ No

Pre-analytical phase of the surface control plan

The pre-analytical phase corresponds to the choices concerning the types of materials and the surfaces to be sampled, the methods and tools for sampling, the periodicity of these as well as the microbiological signals to be sought.

16. Is there a person or team dedicated to microbiological monitoring of the production environment?

- ◯ Yes, integrated into the quality department
- ◯ Yes, integrated with another service
- ◯ No
- ◯ Other : ____________

17. The main purpose of your microbiological monitoring of the environment is to

- ☐ Validate cleaning and disinfection procedures
- ☐ Monitor the microbial flora in your plant, in order to detect abnormal variations
- ☐ Detection of pathogenic microorganisms
- ☐ Other : ____________

18. Are there procedures for microbiological environmental monitoring before production?

- ◯ Yes
- ◯ No

19. Are there procedures for microbiological monitoring of the environment during production?

- ◯ Yes
- ◯ No

20. Are there procedures for microbiological monitoring of the environment at the end of production?

- ◯ Yes
- ◯ No

21. What are the metabolites and compounds sought during the monitoring procedures of the production environment?

- ☐ Protein
- ☐ Sugars
- ☐ ATP
- ☐ Any
- ☐ Other : ____________

22. What microorganisms are sought for during production environment monitoring procedures?

- [ ] Aeromonas hydrophila
- [ ] Bacillus cereus
- [ ] Campylobacter jejuni
- [ ] Clostridium perfringens
- [ ] Escherichia coli
- [ ] Listeria monocytogenes
- [ ] Listeria spp.
- [ ] Salmonella spp.
- [ ] Shigella
- [ ] Staphylococcus aureus
- [ ] Vibrio parahaemolyticus
- [ ] Yersinia enterocolitica
- [ ] Total coliforms
- [ ] Enterobacteriaceae
- [ ] "Total" flora
- [ ] Total aerobic mesophilic flora 30 ° C
- [ ] Yeasts
- [ ] Molds
- [ ] Aerobic spores
- [ ] Other mesophilic aerobes (including bacteria, yeasts and molds)
- [ ] Other : ____________

23. If the presence of biofilms is monitored, could you cite the method?

Your answer

24. Approximately how many monitoring points have you identified in your factory?

Your answer

25. How many monitoring points are taken during a monitoring procedure, before the start of production?

Your answer

26. How many monitoring points are taken during a monitoring procedure, during production?

Your answer

27. What is the frequency of microbiological surveillance actions, before the start of production?

- ○ Several times a day
- ○ Once a day
- ○ Once a week
- ○ Once a month
- ○ Not concerned
- ○ Other : ____________

29. What is the frequency of microbiological surveillance actions during production?

- ◯ Several times a day
- ◯ Once a day
- ◯ Once a week
- ◯ Once a month
- ◯ Not concerned
- ◯ Other : ____________

30. What is the frequency of microbiological monitoring actions at the end of production?

- ◯ Several times a day
- ◯ Once a day
- ◯ Once a week
- ◯ Once a month
- ◯ Not concerned
- ◯ Other : ____________

31. Are the monitoring points only carried out in areas where the product is highly vulnerable to contamination?

- ◯ Yes
- ◯ No
- ◯ Not concerned

31. Are the monitoring points only carried out in areas where the product is highly vulnerable to contamination?

- ◯ Yes
- ◯ No
- ◯ Not concerned

32. What are the sampling tools for the detection of microorganisms and metabolites?

- ☐ Contact gel box
- ☐ Agar blade
- ☐ Agar Film
- ☐ Swab
- ☐ Sponge
- ☐ Tape
- ☐ Cloths
- ☐ Compresses
- ☐ Pedisacs
- ☐ Other : ____________

Analytical phase

The analytical phase, which takes place in the laboratory, aims to detect the selected microbiological signals.

33. Who is in charge of the analysis of the samples?

- [ ] Internal laboratory
- [ ] External lab
- [ ] The direct debit operator
- [ ] Other : ______

34. What are the methods of analysis used?

- [ ] Cultivation methods
- [ ] Molecular methods
- [ ] Immunological methods
- [ ] Other : ______

Post-analytical phase

The post-analytical phase concerns the interpretation of the results and the implementation of an action plan.

35. Are the interpretation of the results based on classes (grids with different contamination thresholds)?

- ◯ Yes
- ◯ No
- ◯ Not concerned

36. If you have classes, can you name them? (not required)

Your answer ______

37. Is there any tracking of results trends?

- ◯ Yes
- ◯ No
- ◯ Not concerned

38. Does the monitoring of trends influence the monitoring plan?

- ◯ Yes
- ◯ No
- ◯ Not concerned

39. In accordance with the EGAlim law (law n ° 2018-938 of October 30, 2018), do you think that there is a criterion that requires reporting to the authorities, apart from pathogenic microorganisms?

- ◯ Yes
- ◯ No
- ◯ Is not pronounced

40. What do you think would help you identify such a criterion? (not required)

Your answer

Project continuation

We thank you for your contribution to this questionnaire, it was essential for the inventory of the monitoring practices of the production environment. We ask you to answer the questions below, concerning the follow-up and the return following your answers:

In order to go further, would you agree to carry out a short interview, in the coming months, to deepen certain points of the questionnaire?

- ☐ Yes, face to face
- ☐ Yes, by videoconferencing (eg Skype, Skype for business, jitsi, etc.)
- ☐ Yes, by phone
- ☐ No

Would you like to attend the webinar on the state of the art of microbiological monitoring of production environments including regulations and methods [access to the recording of the webinar of November 4]?

- ◯ Yes
- ◯ No

Would you like to attend the restitution of anonymized results obtained following this questionnaire?

- ◯ Yes
- ◯ No

If you wish to participate in the interview, the Webinar and / or the presentation of the results, please enter your email:

Your answer
